# Ligand-activated PPARδ expression promotes hepatocellular carcinoma progression by regulating the PI3K-AKT signaling pathway

**DOI:** 10.1186/s12967-022-03288-9

**Published:** 2022-02-12

**Authors:** Wei Han, Nan Wang, Rui Kong, Wen Bao, Jie Lu

**Affiliations:** 1 Department of Gastroenterology, Shanghai Tenth People’s Hospital, Tongji University School of Medicine, Shanghai, China; 2 Department of Gastroenterology, Gongli Hospital of Shanghai Pudong New Area, Shanghai, China

**Keywords:** PPARδ, HCC, Prognosis, PI3K-AKT

## Abstract

**Background:**

Peroxisome proliferator-activated receptor-beta/delta (PPARδ) was considered as the key regulator involved in the evolution of various tumors. Given that PPARδ potential role in hepatocellular carcinoma (HCC) is still obscure, we comprehensively assessed its expression pattern, prognosis, functions and correlation with tumor microenvironment in HCC using public database data and in vitro studies.

**Methods:**

Transcriptional data and clinical data in the TCGA and GEO database were analyzed in R software. Quantitative real-time polymerase chain reaction (qRT-PCR), western blotting and immunohistochemistry were used to detect the expression level of related RNA and proteins. The malignant biological characteristics were explored by cell counting Kit-8 (CCK8), 5-Ethynyl-2ʹ-deoxyuridine (EdU) assay and wound healing assay.

**Results:**

Our results illustrated that PPARδ expression was significantly higher in HCC tissues and HCC cell lines. Elevated expression of PPARδ suggested poor clinical staging and prognosis in HCC. Ligand-activated PPARδ expression promoted the proliferation and invasion of HCC cells via PDK1/AKT/GSK3β signaling pathway. The expression of PPARδ was closely related to the HCC tumor microenvironment.

**Conclusions:**

PPARδ plays an important part in HCC progression, penetrating investigation of the related regulatory mechanism may shed light upon further biological and pharmacological value.

## Background

Hepatocellular carcinoma (HCC) has become an important malignant disease threatening the people health around the world. In the latest global cancer statistics report in 2020, the incidence of HCC was 4.7% (the seventh place), and the mortality rate was 8.3% (the second place) [1]. Although the popularization of the hepatitis B vaccine and the development of multiple therapeutic methods enhance the curing possibility, the number of deaths related to HCC remains massive, since vague symptoms and individual specificity may respectively lead to the difficulty in diagnosis and different sensitivity to drug [2, 3]. Therefore, more prognostic biomarkers and therapeutic targets need to be dug up.

Peroxisome proliferator-activated receptors (PPARs) are members of the ligand-activated nuclear transcription factor superfamily. The three isoforms of PPARα (PPARA), PPARδ/β (PPARD), and PPARγ (PPARG) are distributed in different organs and participate in a variety of energy metabolism processes [4, 5]. Activated by endogenous ligands, including fatty acids and derivatives or synthetic modulators, they form heterodimers with retinol X receptors (RXR), which bind to PPAR response elements (PPRE) upstream of the target gene promoter and finally regulate the transcription of target genes [6, 7]. There has been increasing recognition of the crucial role they played in tumor progression. PPARα and PPARγ can affect the development of malignant tumors by regulating survival, metastasis, energy metabolism, immune response, and cell stemness maintenance, which has been reported in various common solid tumors including colon cancer, lung cancer, breast cancer, hepatocellular carcinoma, and so on [8–13]. PPARδ is widely expressed in the human body but is mainly highly expressed in skeletal muscle and macrophage, which acts as a vital regulatory factor in fatty acid oxidation, keratinocyte differentiation and wound healing [14]. PPARδ has been found to be differentially expressed in several major human cancers including colorectal cancer, gastric cancer and prostate cancer, and may function as oncogene or tumor suppressor through diverse mechanisms [15–17]. In most of the reported literature, activation or high expression of PPARδ has been demonstrated to be related to cell proliferation and tumor growth [18]. Additionally, PPARδ was considered to be a key regulator involving tumor metastasis, which strongly enhanced angiogenesis, epithelial-mesenchymal transition (EMT), invasion and migration [19, 20]. Currently, there have been controversial opinions regarding its relationship with tumor development due to the specificity of tumor types. Given the limited research on the biological functions of PPARδ in HCC, multi-dimensional analysis was used to investigate the expression pattern, prognostic value and biological function of PPARδ as well as the relationship with tumor microenvironment (TME) based on resources from public databases and in vitro studies.

## Materials and methods

### Microarray data acquisition and differential expression analysis

The transcriptome expression profiles of HCC were downloaded from The Cancer Genome Atlas (TCGA) and Gene Expression Omnibus (GEO) database. We performed differential mRNA expression analysis with “ggpubr” R package in R software (Version 4.0.2).

### Cell culture

Three HCC cell lines, Bel 7402, SMMC-7721, HepG2 and human liver cell line LO2 were obtained from the Cell Bank of Type Culture Collection of the Chinese Academy of Sciences (Beijing, China). Four cell lines were cultured in high glucose Dulbecco’s modified Eagle’s medium (DMEM, Thermo Fisher Scientific, Waltham, MA, USA) with 10% fetal bovine serum (FBS, Thermo Fisher Scientific, Waltham, MA, USA). The cells were cultured in a 37 °C, 5% CO_2_ incubator and were sub-cultured when the cell density reached 90%.

Cells were exposed to different concentrations of PPARδ agonist GW501516 (Topscience Co., Ltd., Shanghai, China) or GW501516 and PPARδ antagonist GSK3787 (Topscience Co., Ltd., Shanghai, China) for 24–48 h. Morphology changes were observed using a phase-contrast microscope and imaged (200×).

### Correlation of clinical characteristics and survival analysis

Clinically relevant data such as age, gender, stage, grade, survival time, and outcome was obtained from TCGA database. The samples were divided into high expression group and low expression group according to the mean value of PPARδ mRNA expression. The relationship between mRNA expression and grade, TNM staging was tested in R software (Version 4.0.2). The Kaplan–Meier (K-M) survival curves were drawn by GraphPad Prism 8. Cox regression analysis was performed using “forestplot” and “rms” R packages in R software (Version 4.0.2).

### Quantitative real-time PCR (qRT-PCR)

Total RNA was extracted from the cell lines using Trizol reagent (Invitrogen, Carlsbad, CA, USA). The RNA was reverse transcribed into cDNA using the transcription kit from Takara Biotechnology (Dalian, China). qRT-PCR was performed in the Applied Bio-systems 7500 Real-Time PCR System using 50 ng of cDNA and a SYBR Green PCR master mix (YEASEN, Shanghai, China). β-actin was used as the internal control gene. The quantitative level of mRNA was evaluated by the 2^^−ΔCt^ relative quantitative method. The primers were as follows:

β-actin:

F: CATGTACGTTGCTATCCAGGC

R: CTCCTTAATGTCACGCACGAT

PPARδ:

F: CAGGGCTGACTGCAAACGA

R: CTGCCACAATGTCTCGATGTC

ANGPTL4:

F: TGGGACGAGATGAATGTCCTG

R: CTGCTGTTCTGAGCCTTGAGTT

### Western blotting (WB) analysis

The total proteins were extracted from the cell lines using RIPA lysis buffer (Invitrogen, USA). Protein concentration was determined with a BCA kit (Kaiji Biology, Nanjing, China). WB was performed as routine. The primary antibodies were β-actin (1:5000, 60008-1-Ig, Proteintech, Chicago, USA), PPARδ (1:500, 10156-2-AP, Proteintech, Chicago, USA), E-cadherin (1:50, ab1416, abcam, Cambridge, UK), Snail (1:1000, 3879, Cell Signaling Technology, Danvers, MA), VEGFA (1:1000, ab1316, abcam, Cambridge, UK), PDK1 (1:1000, 3062, Cell Signaling Technology, Danvers, MA), p-PDK1(1:500, 3438, Cell Signaling Technology, Danvers, MA), AKT (1:1000, C67E7, Cell Signaling Technology, Danvers, MA), p-AKT (1:500, 4060, Cell Signaling Technology, Danvers, MA), GSK3β (1:1000, 22104-1-AP, Proteintech, Chicago, USA), p-GSK3β (1:500, 5558, Cell Signaling Technology, Danvers, MA), Cyclin D1 (1:500, 2978, Cell Signaling Technology, Danvers, MA) and the secondary antibody was horseradish peroxidase-conjugated anti-rabbit (1:500, 7074, Cell Signaling Technology, Danvers, MA) or anti-mouse IgG (1:500, 7076, Cell Signaling Technology, Danvers, MA). The relative band intensities of PPARδ were calculated using the Odyssey Two-color Infrared Laser Imaging System (Li-Cor, Lincoln, NE).

### Tissue samples acquisition and immunohistochemical analysis

Paraffin biopsies of HCC tissues and paracancerous tissues from 4 patients were obtained from the Shanghai Tenth People’s Hospital. This study was approved by the human study ethics committees at Shanghai Tenth People’s Hospital. All patients provided written informed consent for participation in the study. Specimens were handled in accordance with legal and ethical regulations.

The paraffin sections were washed twice in xylene, and then soaked in anhydrous ethanol, anhydrous ethanol, 85% ethanol, 75% ethanol, and distilled water in sequence. Next, the sections were added with citric acid antigen repair solution, baked in the microwave oven and washed with phosphate buffered saline (PBS) after cooling. 3% hydrogen peroxide solution was added to the slices to block peroxidase, incubated in the dark at room temperature and washed with PBS. After blocking the sections with 3% bovine serum albumin (BSA), drop the primary antibody prepared in an appropriate proportion onto the sections and incubate overnight at 4 °C in a humidified box. The primary antibody that we used was PPARδ (1:200, 10156-2-AP, Proteintech, Chicago, USA). The secondary antibody diluent of the corresponding species of the primary antibody was added dropwise to the sections, and incubated at 37 °C. Next, the sections were stained with DAB color developing solution and hematoxylin, respectively. Finally, the sections were dehydrated in 75% ethanol, 85% ethanol, anhydrous ethanol, anhydrous ethanol, n-butanol, xylene, and sealed with neutral gum after drying. The sections were observed by optical microscopy. The integrated optical densities (IODs) of the PPARδ were calculated using Image-Pro Plus software 6.0 (Media Cybernetics, Silver Spring, MD, USA).

### Cell counting kit-8 (CCK8) assay

Two HCC cell lines, Bel 7402 and SMMC-7721, were plated in 96-well plates at a density of 2 × 10^4^ cells/ml. After the cells were incubated for 24 h, the medium was replaced with DMEM containing PPARδ agonist GW501516 or GW501516 and PPARδ antagonist GSK3787. After 48 h of drug intervention, the cells were treated with the CCK8 kit (YEASEN, Shanghai, China) and detected of the absorbance (OD) values at 450 nm using microplate reader (Synergy H4, BioTek, Winooski, VT, USA).

### 5-Ethynyl-2′-deoxyuridine (EdU) assay

Bel 7402 and SMMC-7721 cells were inoculated into 6-well plates containing glass microscope slides. GW501516 or GW501516 and GSK3787 were added to each cell line after 24 h. After 48 h of drug action, the cells were washed with PBS, fixed with 4% paraformaldehyde, and incubated with glycine. Add 100 μl of penetrant (0.5% TritonX-100 in PBS) to each well and incubate on a rocker device for 10 min, then add 100 ul of 1X Apollo® staining reaction solution and incubate for 30 min at room temperature and protected from light. Next, the cells were washed sequentially with 100 μl of penetrant and 100 μl of methanol. The removed slides were washed three times with PBS in a rocker device. After that, the DAPI dye solution was added dropwise to stain the nucleus at room temperature for 10 min, kept in dark place. Finally, the slides were mounted with anti-fade mounting medium. Fluorescent microscopy was used for microscopic observation and image collection.

### Wound healing assay

Bel 7402 and SMMC-7721 were inoculated on a 6-well plate, scratched with a 200 μl pipette tip when the cells were 80–90% confluent, washed with PBS and then cultured in 1% low concentration serum containing GW501516 or GW501516 and GSK3787. The results were analyzed using Image J software, the healing rate was quantified by calculating the gap size.

### Immunological analysis

CIBERSORT tool can calculate the immune cell compositions using gene expression profiles [21]. We measured the infiltration of 22 types of immune cells in HCC samples by CIBERSORT, and further evaluated their relationship with PPARδ expression. All processes were carried out in R software (Version 4.0.2).

### Statistical analysis

Student’s t-test was used for two-group comparisons, one-way ANOVA was used for multigroup comparisons. The Kaplan–Meier survival curves were drawn based on log rank test. Univariate and multivariate analysis were performed using the Cox regression analysis model. Spearman test was used to verify the correlation between 22 kinds of immune cells content and gene expression. All data analysis was performed in SPSS 21.0 software. The criterion of significant difference is defined as *p* < 0.05.

## Results

### Elevated expression of PPARδ in HCC tissue and HCC cell

**Fig. 1:**
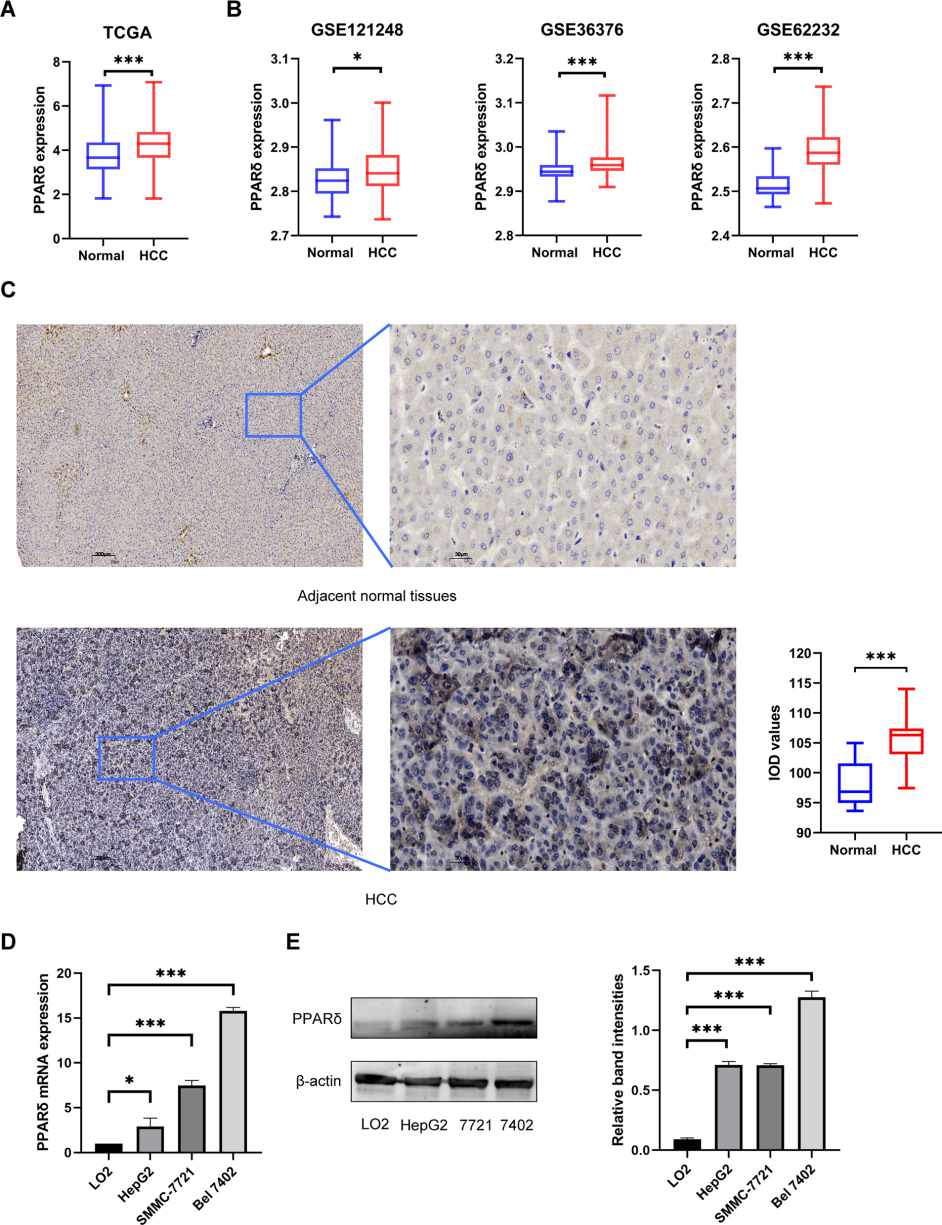
Differential expression of PPARδ in HCC tissue and HCC cell line. Bioinformatics analysis suggested that PPARδ expression was significantly (*p* < 0.001) higher in HCC than in normal tissues based on TCGA database and GEO databases (**A**, **B**). Immunohistochemical analysis of 4 HCC tissues showed that PPARδ was highly expressed in HCC tissues but was weakly positive in adjacent tissues (*p* < 0.001) (**C**). qRT-PCR results suggested the mRNA expression of PPARδ in Bel 7402 (*p* < 0.001), SMMC-7721 (*p* < 0.001) and HepG2 cells (*p* < 0.05) was significantly higher than that of LO2 cell (**D**). WB results suggested that the protein expression of PPARδ was increased in Bel 7402 (*p* < 0.001), SMMC-7721 (*p* < 0.001) and HepG2 cells (*p* < 0.001) compared with LO2 cell (**E**)

We acquired 424 transcriptome files (374 HCC samples and 50 normal samples) from the TCGA database and 631 transcriptome files (391 HCC samples and 240 normal samples) from the GEO database. The results suggested that PPARδ expression was significantly higher (*p* < 0.05) in HCC tissues than in normal tissues at the transcriptional level (Fig. 1A and B). Immunohistochemical analysis showed that PPARδ was highly expressed in HCC tissues but was weakly positive in adjacent tissues (*p* < 0.001) (Fig. 1C). In in vitro experiments, the expression of PPARδ in Bel 7402, SMMC-7721 and HepG2 cells was significantly higher than that of LO2 cells, which was confirmed by qRT-PCR and WB (Fig. 1D and E).

### Association of PPARδ expression and clinical characteristics

**Fig. 2:**
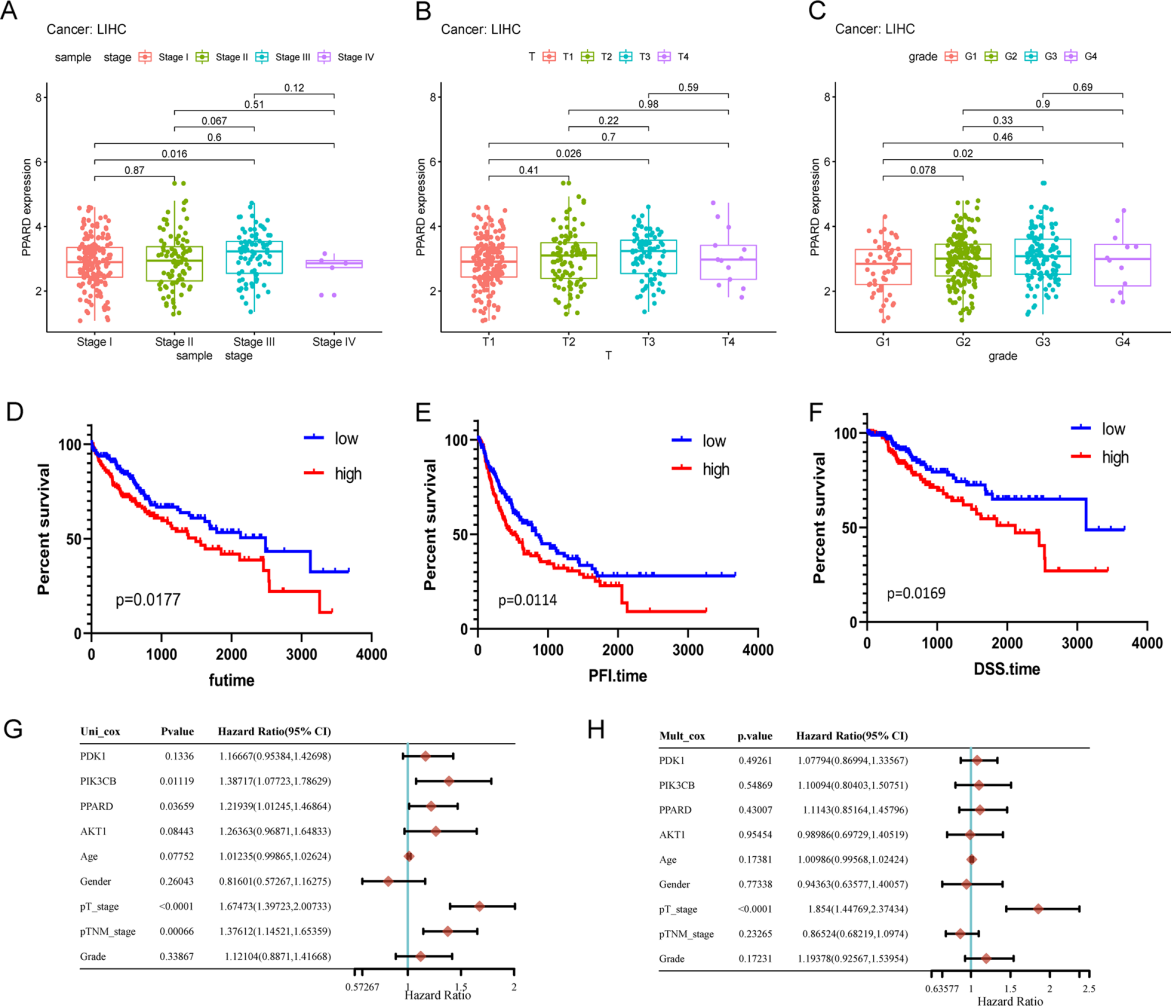
Association of PPARδ expression and clinical characteristics. The expression of PPARδ increased gradually with the progress of clinical stage, T stage and grade (**A**–**C**). The low PPARδ expression group had significantly higher over survival (OS) rate (*p* = 0.0177) (**D**), PFS (progression free survival) rate (*p* = 0.0114) (**E**) and DSS (disease specific survival) rate (*p* = 0.0169) (**F**) than that of high PPARδ expression group. Cox regression analyses suggested that PPARδ, PI3K, PDK1, AKT1 were risk factors for poor prognosis of HCC (HR > 1) (**G**, **H**)

The clinical files of 377 patients downloaded from the TCGA database, including age, sex, clinical stage, lymph node status, distant metastasis, pathology classification, were presented in Table 1. As shown in Fig. 2, the expression level of PPARδ was elevated while the clinical staging and grading of tumor progressed (Fig. 2A, B and C). Among them, the number of stage IV, T4, and G4 patients may be too small to be of statistical value. These results revealed that PPARδ may affect the progression of HCC. Kaplan–Meier survival curves were drawn to investigate the relationship between the expression of PPARδ and various clinical outcomes. High PPARδ expression predicted poorer over survival (OS) (Fig. 2D), progression free survival (PFS) (Fig. 2E) and disease specific survival (DSS) (Fig. 2F) in patients with HCC. Besides, Cox regression analyses suggested that PPARδ, PI3K, PDK1, AKT1 were risk factors for poor prognosis of HCC (HR > 1) (Fig. 2G and H).

**Table 1:** Clinicopathological characteristics of patients with HCC, retrieved from the TCGA database

Category	Variables	Value	Percentage (%)
Sex	Male	255	67.64
	Female	122	32.36
Age (years)	Range	16–90	/
	Median	59.45	/
Stage	I	175	6.42
	II	87	23.08
	III	86	22.81
	IV	5	1.33
	Unidentified	24	6.36
T stage	T1	185	49.07
	T2	95	25.20
	T3	81	21.49
	T4	13	3.45
	TX	3	0.79
Lymph node status	N0	257	68.17
	N1	4	1.06
	Nx	116	30.77
Metastatic	M0	272	72.75
	M1	4	1.06
	MX	101	26.19
Grade	G1	55	14..59
	G2	180	47.75
	G3	124	32.89
	G4	13	3.45
	Unidentified	5	1.32

### PPARδ promoted HCC cell proliferation

**Fig. 3:**
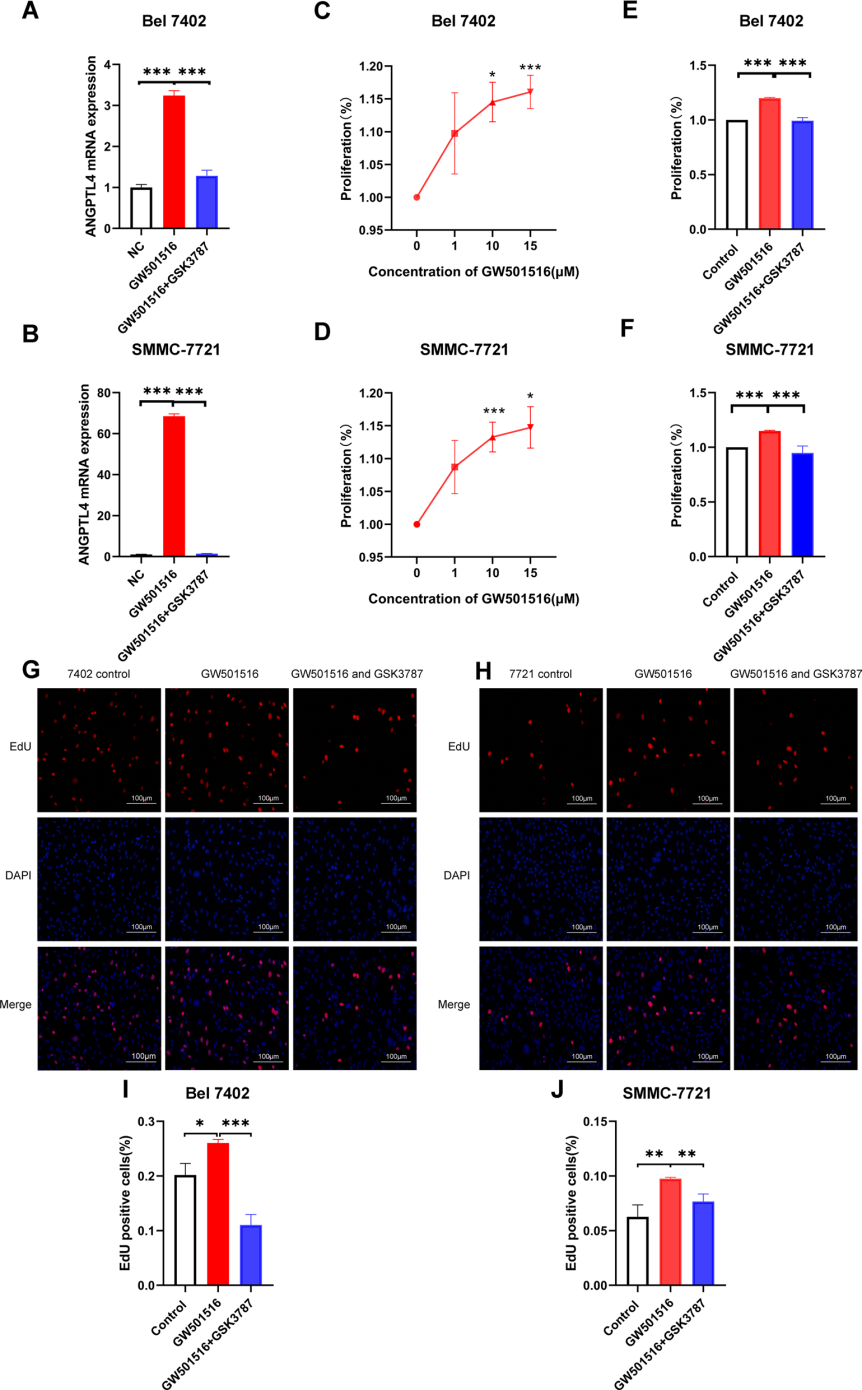
Effect of PPARδ on proliferation of HCC cell. PPARδ agonist GW501516 increased mRNA expression of PPARδ target gene Angptl4. while antagonist GSK3787 reversed its up-regulated expression (**A**, **B**). The CCK8 assay results revealed that the agonist promoted HCC cells proliferation in a dose-dependent manner (**C**, **D**) and GSK3787 reversed the proliferation effect of GW501516 (**E**, **F**). EdU staining assay results showed that the intervention of GW501516 increased the ratio of proliferating cells, and GSK3787 reversed the proliferation effect of GW501516 in Bel 7402 cell line (**G**, **I**) and SMMC-7721 cell line (**H**, **J**)

The cell proliferation ability was evaluated using the CCK8 kit and EdU assay. GW501516 is an effective and highly specifc PPARδ agonist [25]. GSK3787 could antagonize ligand-induced expression of PPARδ-dependent genes, but it does not antagonize basal expression of these genes [26]. GW501516 increased mRNA expression of PPARδ target gene Angptl4 while GSK3787 reversed its up-regulated expression, which suggested that GW501516 activated the expression of PPARδ and GSK3787 antagonized the activation (Fig. 3A and B). The HCC cell lines, Bel 7402 and SMMC-7721, were treated with GW501516 (1 μM, 10 μM, 15 μM) for 48 h. The CCK8 results revealed that PPARδ agonist promoted HCC cells proliferation in a dose-dependent manner (Fig. 3C and D), and GSK3787 reversed the proliferation effect of GW501516 (Fig. 3E and F). In the EdU staining assay, the intervention of GW501516 (15 μM) significantly increased the ratio of proliferating cells compared to control groups, and GSK3787 (10 μM) reversed the effect in Bel 7402 cell line (Fig. 3G and I) and SMMC-7721 cell line (Fig. 3H and J). Taken together, ligand-activated PPARδ enhances the proliferative capacity of HCC cells.

### PPARδ promoted HCC cell migration, EMT and angiogenesis

**Fig. 4:**
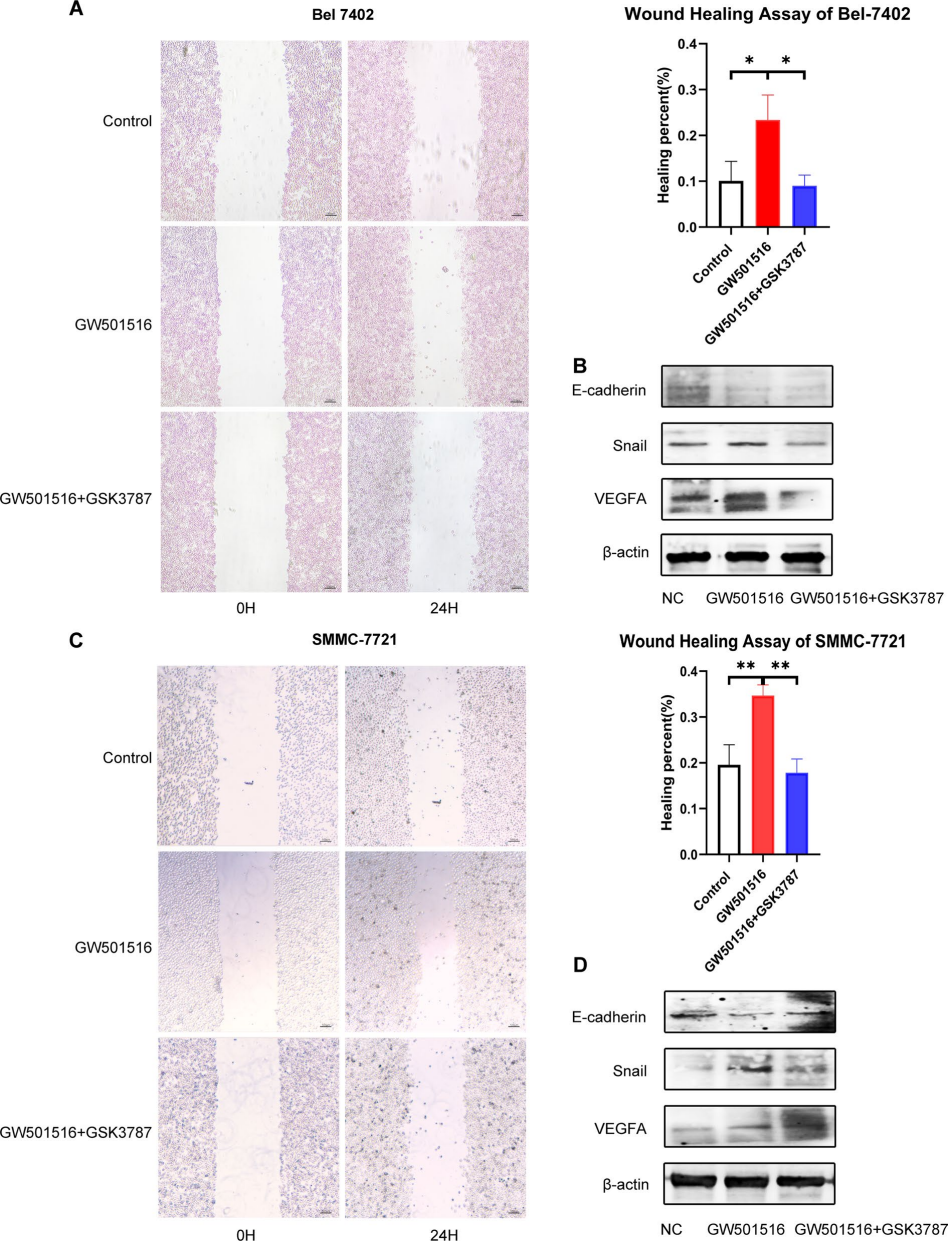
Effect of PPARδ on migration, EMT and angiogenesis of HCC cell. PPARδ agonist GW501516 promoted HCC cells migration and PPARδ antagonist GSK3787 reversed the effect of GW501516 (**A**, **C**). GW501516 reduced the expression of E-cadherin, increased the expression of Snail and VEGFA, the antagonist abolished the effect (**B**, **D**)

Cell migration was measured using wound healing assay. The healing percent of the GW501516 treatment group was greater than that of the control group and GSK3787 abolished the effect (Fig. 4A and C).

We examined whether PPARδ activation had a positive effect on EMT and angiogenesis by measuring the expression of EMT-related markers and VEGFA. GW501516 reduced the expression of E-cadherin, increased the expression of Snail and VEGFA, the antagonist abolished the expression changes (Fig. 4B and D). Taken together, ligand-activated PPARδ facilitate migration, EMT, and angiogenesis in HCC cells.

### PPARδ partially activated PI3K-AKT signaling pathway

**Fig. 5:**
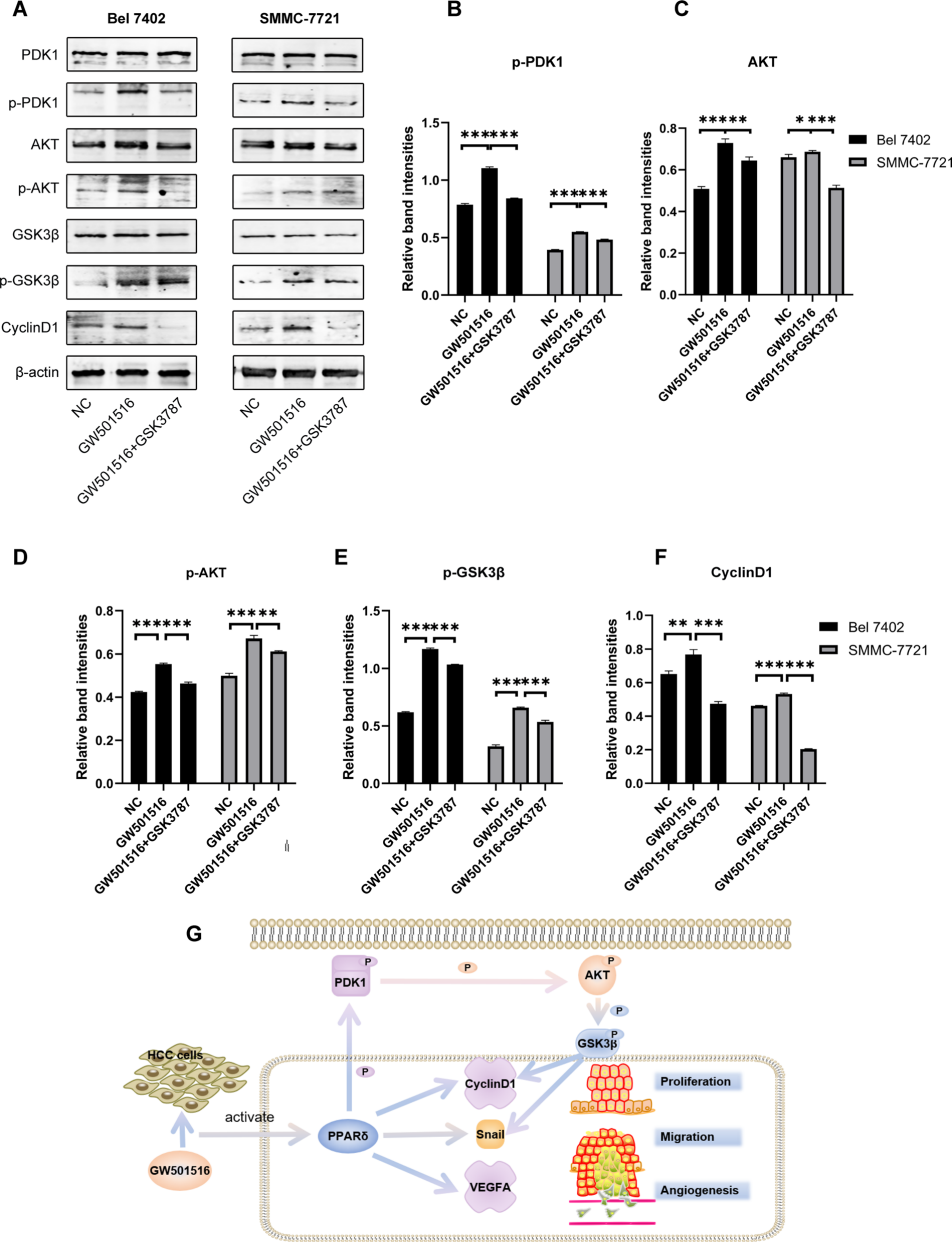
PPARδ promoted HCC progression by regulating the PI3K-AKT signaling pathway in HCC cell (**A**). PPARδ agonist GW501516 increased the expression of p-PDK1 (**B**), AKT (**C**), p-AKT (**D**), p-GSK3β (**E**) and Cyclin D1 (**F**) in Bel 7402 and SMMC-7721 cell, the antagonist abolished the effect. Schematic diagram of the role of PPARδ/PDK1/AKT/GSK3β signaling pathway in HCC progression (**G**)

Literature has shown that the carcinogenesis of PPARδ was related to the activation of the PI3K-AKT pathway [22, 23]. Therefore, we detected the expression of proteins related to the PI3K-AKT pathway in different treatment groups of HCC cells. The results revealed that GW501516 intervention up-regulated the expression of p-PDK1, AKT, p-AKT, and its downstream effector p-GSK3β and Cyclin D1. GSK3787 had an antagonistic effect on these proteins (Fig. 5A–F). All these data indicate that PPARδ could partially activated the PI3K-AKT pathway (Fig. 5G).

### Immune relevance of PPARδ

**Fig. 6:**
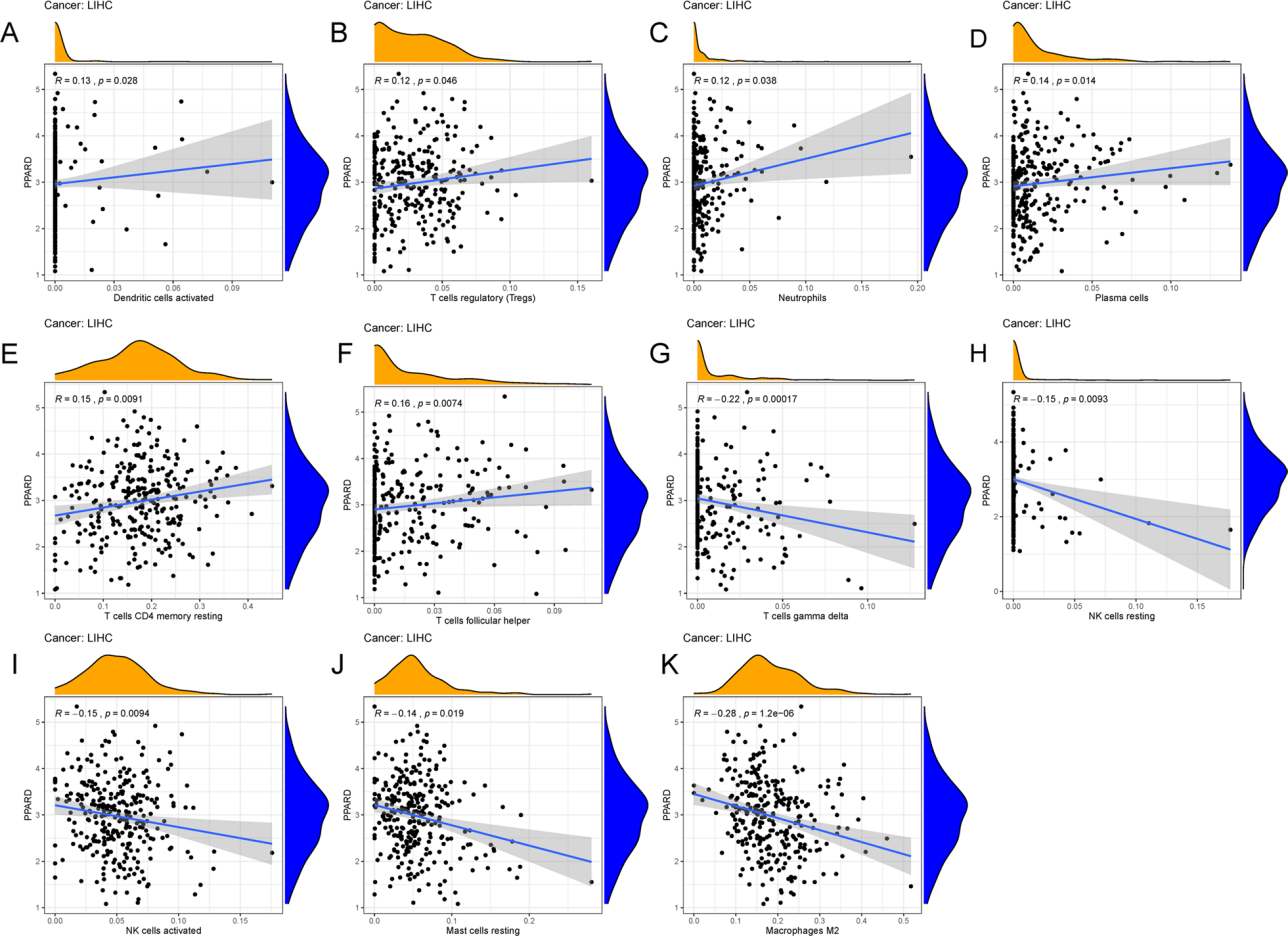
The relationship between the expression level of PPARδ and immune cell content. PPARδ expression had a positive correlation with the infiltration of dendritic activated cells (**A**), regulatory T cells (**B**), neutrophils (**C**), plasma cells (**D**), CD4 memory resting T cells (**E**), follicular helper T cells (**F**) and a negative correlation with the infiltration of gamma delta T cells (**G**), resting NK cells (**H**), activated NK cells (**I**), resting mast cells (**J**) and M2 macrophages (**K**)

In order to explore the role of PPARδ in the TME, we calculated the infiltration of immune cells in HCC samples by means of CIBERSORT tool. Next, we analyzed the relationship between the expression level of PPARδ and immune cell content. The analysis revealed that PPARδ expression had a positive correlation with the infiltration of dendritic activated cells (Fig. 6A), regulatory T cells (Fig. 6B), neutrophils (Fig. 6C), plasma cells (Fig. 6D), CD4 memory resting T cells (Fig. 6E), follicular helper T cells (Fig. 6F) and a negative correlation with the infiltration of gamma delta T cells (Fig. 6G), resting NK cells (Fig. 6H), activated NK cells (Fig. 6I), resting mast cells (Fig. 6J), and M2 macrophages (Fig. 6K).

## Discussion

The role of PPARδ as oncogenic factor or tumor suppressor factor is still controversial, which is influenced by tumor types and experimental methods [15, 17, 20, 24–26]. In gastric cancer, researchers found that overexpression of PPARδ in the villin promoter of mice quiescent gastric progenitor cells promoted the occurrence of spontaneously invasive gastric adenocarcinomas [16]. In addition, they found that the expression of PPARδ was significantly increased in human gastric tissues and was associated with poor prognosis [16]. In colon cancer, it was revealed that the expression of PPARδ in human colon cancer tissues was significantly higher than that of adenoma polyps and normal intestinal mucosa. Its cancer-promoting mechanism has also been further explored in colorectal tumor cell lines and colon cancer mouse models [17, 27–29]. Similarly, some teams have reached the opposite conclusion [30, 31]. The latest research pointed out that PPARδ promoted liver metastasis of colon cancer by inducing the expansion of colonic stem cells [32]. In melanoma, study has shown that PPARδ activation modulated the migration and invasion of melanoma cells by up-regulating Snail expression [33]. In this study, analysis based on the TCGA and GEO transcriptome data revealed a significantly increased expression of PPARδ in HCC compared with normal liver tissues. What's more, the immunohistochemical analysis of the HCC tissues in our medical facility demonstrated its overexpression at the protein level. The significant clinical relevance of PPARδ, including TNM stage and pathology classification, was also disclosed. Especially, K-M survival curves for OS, DSS, PFS and Cox analysis suggested that high expression of PPARδ displayed a significant unfavorable prognosis. The high expression of PPARδ in HCC cells was also verified. In cell function experiments, we proved that PPARδ can promote the proliferation and migration of HCC cells. Consequently, digging into the PPARδ related mechanisms may contribute to give full play to its biological and pharmacological effect.

EMT had a positive effect on tumor invasion and distant metastasis, in which epithelial cells lost intercellular adhesion and obtained mesenchymal properties [34, 35]. Studies have shown that the transcription factor Snail was one of the main EMT-inducing factors in HCC and was related to the prognosis of HCC [36]. Snail binded to three E-boxes in the human E-cadherin promoter, thereby suppressed transcription of E-cadherin [37, 38] Therefore, we determined the expression of these two EMT markers. The results revealed that activation of PPARδ up-regulated the expression of the epithelial marker Snail and down-regulated the expression of the mesenchymal marker E-cadherin. The “angiogenic switch” effect of PPARδ has been verified by a large amount of literature [18]. In our study, we also found that HCC cells treated with agonist expressed more VEGFA. Taken together, PPARδ may induce EMT by regulating Snail expression and promote angiogenesis through up-regulation of VEGFA expression.

The PI3K-AKT pathway was a key regulatory center that regulated cell growth, metabolism, proliferation, survival, transcription and protein synthesis. Activated PI3K catalyzed the 3-hydroxyl phosphorylation of PIP2 to generate PIP3, which acted as a second messenger to recruit PDK1 and AKT proteins to the plasma membrane. PDK1 can phosphorylate the threonine (Thr^308^) of the AKT protein, and the additional phosphorylation of mTORC2 at the c-terminal domain of serine 473 (Ser^473^) resulted in complete activation of AKT. The activated AKT will further activate downstream signal pathways [39, 40]. Currently, PI3K-AKT pathway have got a major focus of attention in tumor progression, inhibitors of this pathway have brought new horizons for targeted tumor therapy [41]. The activation of PI3K-AKT pathway was associated with the progression and metastasis of HCC [42]. One study demonstrated that PPARδ promoted proliferation and inhibited apoptosis through the PI3K-AKT pathway in non-small cell lung cancer [22]. Another study also revealed that PPARδ/PDK1/PTEN/AKT/GSK3β/Cyclin D1 pathway was involved in the proliferation of keratinocytes in cholesteatoma [23]. Therefore, we detected the expression of PI3K-AKT pathway related markers in agonist-intervened HCC cells. We could find that the expression of p-PDK1, AKT, p-AKT, and the targeted effector molecule p-GSK3β and Cyclin D1 has increased. PDK1 was identified as a target gene for PPARδ [43, 44]. Phosphorylated PDK1 can promote the activation of AKT, thereby inhibiting the function of GSK3β through phosphorylation at Ser 9. The inactivation of GSK-3β abolished its degrading effect on cyclin D1, and the expression of cyclin D1 was up-regulated [45]. Besides, the suppression of GSK3β induced by AKT could increase the stability of Snail and promote the occurrence of EMT [46]. In summary, PPARδ may promote HCC progression by partially activating the PI3K-AKT pathway. However, more detailed regulatory network needs further experimental exploration.

It is well known that not only the tumor cells themselves but also the TME composed of stromal cells, immune cells, extracellular matrix (ECM), cytokines, chemokines, growth factors, and enzymes hold an important role in tumor development. We further analyzed the relationship between the expression of PPARδ and the content of infiltrating immune cells in HCC tissues. Among the immune cells fighting tumors, effector T cells and NK cells play a major role. However, metabolic changes in the TME impede the maintenance of T cell normal function, leading to the formation of immune tolerance. Literature has affirmed that PPARα and PPARδ could enhance the persistence of memory CD8 + T cells by targeting the fatty acid oxidation pathway, thereby improving the efficacy of adoptive cell therapy [47]. Regarding NK cells, a recent study indicated that obesity induced PPAR-driven lipid accumulation in NK cells, leading to complete “paralysis” of cellular metabolism and trafficking. Targeting PPARα/δ may restore NK cells function and cytotoxicity [48]. Unlike effector T cells, Tregs cells maintained their active proliferative state through the uptake of lactic acid [49]. Recently, a study proposed the hypothesis that CD36 acted on the PPARδ pathway to fine-tune mitochondrial fitness and thus programed Treg cells to adapt to the lactic acid-enriched TME. Targeting this metabolic pathway to deplete Treg cells showed great antitumor therapeutic potential [50]. In our immune relevance analysis, the expression level of PPARδ was correlated with the infiltration of CD4 + T cells, Treg cells and NK cells, which gives us a hint to thoroughly explore the value of PPARδ pathway in T cell and NK cell metabolism about HCC. M2 polarization of tumor-associated macrophages (TAM) was considered as a marker of tumor progression in HCC [51]. Similarly, PPARδ was believed to be an important downstream regulator for the alternative activation of TAM in HCC caused by SIRT4 gene silencing [52]. Our research suggested that PPARδ expression was significantly related to macrophage subtypes. Taken together, the expression of PPARδ had a close correlation with several immune cells in HCC tumor microenvironment.

There are some limitations in our study. First, our hypothesis obtained from in vitro experiments lacks support from in vivo results. Second, the composition of cells in HCC tissues are complex, other cellular components in the TME, such as immune cells and stromal cells, are equally critical to tumor progression. our study only verified the regulatory role of PPARδ in HCC cells. Therefore, further exploration is badly required.

## Conclusion

In summary, our results illustrated that PPARδ expression was significantly higher in HCC tissues compared to normal liver tissues and was related to progression and poor prognosis of HCC. PPARδ has been shown increased expression in HCC cells and promoted the proliferation and invasion of HCC cells via PDK1/AKT/GSK3β signaling pathway. Further, the level of PPARδ expression was correlated with the infiltration of various immune cells.

## Data Availability

The transcriptome data and clinical data in the study are available at the following hyperlinks: 1.The Cancer Genome Atlas, (TCGA, https://tcga-data.nci.nih.gov/tcga/); 2.Gene Expression Omnibus, (GEO, http://www.ncbi.nlm.nih.gov/geo/).

## Abbreviations

HCC: Hepatocellular carcinoma
PPARα: Peroxisome proliferator activated receptor alpha
PPARγ: Peroxisome proliferator activated receptor gamma
PPARδ: Peroxisome proliferator activated receptor delta
AKT: Protein kinase B
PI3K: Phosphoinositide 3-kinase
PDK1: Pyruvate dehydrogenase kinase 1
GSK3β: Glycogen synthase kinase 3 beta
TCGA-LIHC: The Cancer Genome Atlas-liver hepatocellular carcinoma dataset
